# Observational study to determine the proportion of first milking colostrum from Scottish dairy herds positive for *Mycoplasmopsis bovis*

**DOI:** 10.1002/vetr.5594

**Published:** 2025-08-26

**Authors:** Alexandra Haggerty, David Bell, Colin Mason, Katharine S. Denholm

**Affiliations:** ^1^ School of Biodiversity One Health and Veterinary Medicine, University of Glasgow Glasgow UK; ^2^ Scotland's Rural College Edinburgh UK; ^3^ Scotland's Rural College Veterinary Services, Dumfries Vet Centre Dumfries UK

## Abstract

**Background:**

*Mycoplasmopsis bovis* causes a range of clinical conditions, including mastitis, arthritis, otitis and bronchopneumonia. Proposed transmission routes include semen, milk, colostrum, aerosol, nose‐to‐nose contact and fomite spread.

**Methods:**

Seventy‐nine composite colostrum samples were collected directly from cows’ teats on 10 farms in the Dumfries and Galloway region of Scotland. The samples were transported on ice to the laboratories at Scotland's Rural College, where they underwent *M. bovis* polymerase chain reaction testing.

**Results:**

Of the 79 samples tested, 78 tested negative for the presence of *M. bovis* DNA and one returned an inconclusive result.

**Limitations:**

Samples were not randomly selected but were instead convenience samples from a cohort of progressive farms. As such, the findings may not be representative of the wider population of Scottish dairy herds.

**Conclusion:**

Results from this work suggest that colostrum as a transmission route for *M. bovis* is less important than other routes, such as nose‐to‐nose contact or fomite spread.

## INTRODUCTION

*Mycoplasmopsis bovis* (formerly *Mycoplasma bovis*) is a pathogenic species of bacteria belonging to the Mollicutes family.^1^ It causes a range of clinical conditions in both adult dairy cattle and calves. In calves, disease processes such as bronchopneumonia, arthritis, otitis and keratoconjunctivitis are common, while in adult cattle, chronic, intractable mastitis can be a feature of *M. bovis* infection.^2^ These disease processes ultimately lead to chronic pain, reduced mobility and decreased milk production, compromising overall animal welfare.^2^ *M. bovis* is present on dairy farms worldwide and can be a risk factor for high antibiotic use, particularly in youngstock.^3^ The UK is no exception.^3^, ^4^

*M. bovis* is characterised by the lack of a cell wall and its ability to evade the host immune system.^5^ These characteristics mean that *M. bovis* can be challenging to treat. Vaccine development has also proven problematic because of the high genetic and antigenic variability of field *M. bovis* strains, although there are now several licensed vaccines available.^6^ Given these challenges, management of *M. bovis* is typically centred on biosecurity and biocontainment measures, with approaches being multifactorial and specific to individual farms.^7^ It is therefore important to determine the relative significance of transmission routes for *M. bovis* to ensure that efforts and resources allocated to infection control at the farm level yield a measurable return on investment.

Purported transmission routes for *M. bovis* include semen, infected milk and colostrum, aerosol, nose‐to‐nose contact and fomite spread.^8^ The relative importance of each of these routes of transmission is largely unknown.^9^ Belgian work by Gille et al.^10^ studied first milking colostrum from 17 farms (beef, dairy, mixed), four of which had recent *M. bovis* infection within the herd (<1 month), as shown by a positive culture or polymerase chain reaction (PCR). In the aforementioned study, the within‐herd prevalence of *M. bovis* in first milking colostrum was determined to be 3.2% (standard deviation: ±4.2%; range: 0‒30%). The prevalence of *M. bovis* in first milking colostrum has not been fully elucidated in the UK. It is hypothesised that colostrum transmission of *M. bovis* from dam to calf may be less important in Scottish dairy herds than other transmission routes, such as nose‐to‐nose contact or fomite spread. The objective of this study was to measure the proportion of colostrum samples that contained *M. bovis* DNA (using PCR testing) from a convenience sample of 10 Scottish dairy herds.

## MATERIALS AND METHODS

Samples were collected as part of a larger prospective, observational study, conducted between March and November 2023 (under the University of Glasgow ethics licence number EA10/23), exploring bacterial contamination of first milking colostrum (see sister paper by Haggerty et al.^11^). Ten dairy farms were conveniently enrolled from the client lists of two commercial veterinary practices in the Dumfries and Galloway region of Scotland. The farms included in the study employed various management systems, such as seasonal calving (both spring and autumn), as well as all‐year‐round calving systems; additionally, both grazing and housed systems were used. Further details on the farm demographics are provided in Table 1.

**TABLE 1:** Information on farm demographics and *Mycoplasmopsis bovis* diagnostic history, as collected by farmer questionnaire, for the 10 Scottish dairy farms enrolled in the study between March and November 2023

Farm ID	Herd size	Proportion of herd sampled (*n*)	Previous *M. bovis* diagnosis	Laboratory testing
Farm 1	180	2.2% (4)	Yes	Calf serum antibody ELISA
Farm 2	750	1.3% (10)	No	N/A
Farm 3	515	1.9% (10)	No	N/A
Farm 4	850	0.7% (6)	Yes	PCR of nasal swabs
Farm 5	1800	0.3% (6)	Yes	Postmortem examination
Farm 6	550	0.9% (5)	Yes	Bulk milk antibody ELISA
Farm 7	450	2.9% (13)	Yes	Bulk milk antibody ELISA, calf serum antibody ELISA
Farm 8	180	5% (9)	Yes	Bulk milk antibody ELISA
Farm 9	150	8% (12)	Yes	Calf serum antibody ELISA
Farm 10	650	0.6% (4)	Yes	Calf serum antibody ELISA and postmortem examination

Farmers were asked to complete a questionnaire detailing their colostrum management protocols with the principal researcher at the time of enrolment, including pertinent farm history regarding *M. bovis* infection and diagnosis (from previous calf blood samples, postmortem examinations and bulk milk testing by the commercial veterinary clinician). All cows and heifers calving within the study period were eligible for enrolment. Farm staff were trained to use a standard operating procedure for composite colostrum sample collection directly from each individual cow's teats into factory clean 50 mL sample containers.

The samples were then stored at ‒18°C before being transported on ice for testing at the external Scotland's Rural College laboratories (SRUC Veterinary and Analytical Services, Pentland Science Park, UK). Briefly, DNA was extracted from samples using the Applied Biosystems MagMAX CORE Nucleic Acid Purification kit, and PCR was conducted using the Applied Biosystems VetMAX *M. bovis* kit (both supplied by ThermoFisher Scientific). On‐farm freezer temperatures were monitored using data loggers throughout the on‐farm storage period (Elitech RC 5+, Elitech). A subset of teat samples (*n* = 79) were selected at random from 152 teat samples collected throughout the trial for testing for the presence of *M. bovis* by PCR. The results were reported semi‐quantitatively as ‘‒’, ‘+’, ‘++’, and ‘+++’ by the laboratory, based on the Ct (cycle threshold) of the sample in each reaction. Ct is defined as the number of cycles required for the fluorescent signal to cross the threshold (i.e., exceed background level). *M. bovis* was considered present if a Ct value of less than 37 was obtained, Ct values between 37 and 40 were inconclusive and *M. bovis* was considered absent if a Ct value was undeterminable. To detect an actual prevalence of 5% *Mycoplasma* species in colostrum with a desired precision of 0.05 and confidence of 95%, 75 colostrum samples were required.

## RESULTS

Table 1 summarises descriptive data for each farm, including herd size and the number of samples tested per farm. Seventy‐eight (98.73%) of the 79 colostrum samples tested were PCR negative for *M. bovis*. One sample (1.27%, 95% confidence interval: 0.032‒6.94) from farm 7 was inconclusive, (Ct value 37.82) suggesting potentially low levels of *M. bovis*.

## DISCUSSION

This is the first known UK study to investigate the proportion of first milking colostrum samples from commercial dairy herds that were PCR positive for *M. bovis*. The nature of the PCR test means that it was not possible to ascertain whether positive colostrum samples contained viable *M. bovis* organisms. PCR testing identifies the presence of pathogen DNA only and provides no information about the infectious nature of the *M. bovis* detected.^12^ Further work is needed to determine the proportion of colostrum samples containing viable *M. bovis*.

In the current study, colostrum samples were frozen at −18°C prior to thawing and testing. Gille et al. examined the effect of freezing on the recovery of *Mycoplasma* from colostrum and concluded that storage of colostrum samples at −18°C likely has little influence on qualitative bacteriological testing for *M. bovis*.^13^

The present study found a low proportion of samples were PCR positive for *M. bovis* (1.27%, *n* = 1/79), with the high Ct value obtained meaning the positive test result is not conclusive. Previous work exploring bulk milk tank samples found that only 1.7% (*n* = 3/181) tested positive for *M. bovis* using PCR.^4^ Similar Belgian work on the prevalence of *M. bovis* in colostrum estimated the overall study population prevalence to be 1.9%, and the within‐herd prevalence to be 3.2%.^10^ An Estonian study on four dairy farms endemically infected with *M. bovis* estimated the proportion of PCR‐positive pooled colostrum samples to be between 1.7% and 4.7%.^14^ The methodology used by Gille et al.^10^ differed slightly from the present study, since the inclusion criteria for the Belgian study required a recent (within 1 month of sampling) diagnosis of *M. bovis* by positive culture or PCR. For context, eight of the 10 farms enrolled in the current work had laboratory diagnosis (e.g., nasal swabs, serology, postmortem examination or culture) in the previous 5‐year period.

### Study limitations

The farms enrolled in the present study may not be representative of the wider population because they are progressive dairy farms with motivated staff willing to collect colostrum samples. Any further work to determine the prevalence of *M. bovis* in first milking colostrum will require sample size calculation. Further work needs a larger sample size and also the use of diagnostic testing approaches other than PCR. Furthermore, previous *M. bovis* diagnosis may be indicative of previously implemented control strategies for *M. bovis*, which may have influenced the proportion of positive colostrum samples, although the insidious nature of the pathogen makes this unlikely. In addition, the shedding pattern of *M. bovis* could also be different in herds that experience any *M. bovis* mastitis as a feature of clinical disease, which none of the enrolled farms reported.^15^

## CONCLUSION

Farmers and veterinary practitioners should be mindful of the multifactorial nature of transmission routes for *M. bovis* infection on dairy farms. The current work suggests that colostrum as a transmission route for *M. bovis* infection of calves may be less important in Scottish dairy herds than other transmission routes such as nose‐to‐nose contact or fomite spread; however, further work is needed.

## AUTHOR CONTRIBUTIONS

Alexandra Haggerty contributed to conceptualisation, project administration, investigation and writing the original draft of the manuscript. Katharine S. Denholm contributed to conceptualisation, methodology, investigation, formal analysis, writing—review and editing and visualisation. Colin Mason and David Bell contributed to conceptualisation, data integrity and writing—review and editing. All the authors had full access to all study data, and they have given final approval to the manuscript.

## CONFLICT OF INTEREST STATEMENT

The authors declare that they have no known competing financial interests or personal relationships that could have appeared to influence the work reported in this paper.

## ETHICS STATEMENT

This study was approved by the University of Glasgow ethics committee (number EA10/23).

## Data Availability

The data that support the findings of this study are openly available from University of Glasgow Enlighten database (https://doi.org/10.5525/gla.researchdata.1970).

## References

[1] Oren A , Garrity GM . Valid publication of the names of forty‐two phyla of prokaryotes. Int J Syst Evol Microbiol. 2021;71(10):005056. 10.1099/ijsem.0.005056 34694987

[2] Dudek K , Szacawa E. *Mycoplasma bovis* infections: occurrence, pathogenesis, diagnosis and control, including prevention and therapy. Pathogens. 2020;9(12):994. 33260865 10.3390/pathogens9120994 PMC7760832

[3] Deeney AS , Collins R , Ridley AM. Identification of *Mycoplasma* species and related organisms from ruminants in England and Wales during 2005‒2019. BMC Vet Res. 2021;17:325. 34641885 10.1186/s12917-021-03037-y PMC8513359

[4] Ireland‐Hughes JEH , Tongue SC , Young K , Mason C. Bulk milk prevalence of *Mycoplasma bovis* in Scottish dairy herds. Cattle Pract. 2022;30(1):36.

[5] Ammar A , Abd El‐Hamid MI , Hashem YM , El‐Malt RMS , Mohamed HM . *Mycoplasma bovis*: taxonomy, characteristics, pathogenesis and antimicrobial resistance. Zagazig Vet J. 2021;49:444‒461.

[6] Dudek K , Szacawa E , Nicholas RAJ . Recent developments in vaccines for bovine mycoplasmoses caused by *Mycoplasma bovis* and *Mycoplasma mycoides* subsp. *mycoides* . Vaccines. 2021;9(6):549. 34073966 10.3390/vaccines9060549 PMC8225212

[7] Woolums AR , Chase CCL. Biosecurity and biocontainment for ruminant respiratory disease. Vet Clin Food Anim Pract. 2025;41(1):39‒54. 10.1016/j.cvfa.2024.11.007 39779448

[8] Haapala V , Vähänikkilä N , Kulkas L , Tuunainen E , Pohjanvirta T , Autio T , et al. *Mycoplasma bovis* infection in dairy herds—risk factors and effect of control measures. J Dairy Sci. 2021;104:2254‒2265 33309344 10.3168/jds.2020-18814

[9] Biesheuvel MM , Ward C , Penterman P , Van Engelen E , Van Schaik G , Deardon R , et al. Within‐herd transmission of *Mycoplasma bovis* infections after initial detection in dairy cows. J Dairy Sci. 2024;107:516‒529. 37709017 10.3168/jds.2023-23407

[10] Gille L , Evrard J , Callens J , Supré K , Grégoire F , Boyen F , et al. The presence of *Mycoplasma bovis* in colostrum. Vet Res. 2020;51:54. 32299498 10.1186/s13567-020-00778-w PMC7164199

[11] Haggerty A , Silva E , Anderson T , Bell D , Mason C , Denholm KS . Identifying critical control points for colostrum contamination in first milking colostrum from Scottish dairy herds. Prev Vet Med. 2025;239:106514. 40112443 10.1016/j.prevetmed.2025.106514

[12] Parker AM , Sheehy PA , Hazelton MS , Bosward KL , House JK. A review of *M* *ycoplasma* diagnostics in cattle. J Vet Intern Med. 2018;32:1241‒1252. 29671903 10.1111/jvim.15135 PMC5980305

[13] Gille L , Boyen F , Van Driessche L , Valgaeren B , Haesebrouck F , Deprez P, et al. Effect of freezer storage time and thawing method on the recovery of *Mycoplasma bovis* from bovine colostrum. J Dairy Sci. 2018;101:609‒613. 29102148 10.3168/jds.2017-13130

[14] Timonen AAE , Autio T , Pohjanvirta T , Hakkinen L , Katholm J , Peterson A , et al. Dynamics of the within‐herd prevalence of *Mycoplasma bovis* intramammary infection in endemically infected dairy herds. Vet Microbiol. 2020;242:108608. 32122612 10.1016/j.vetmic.2020.108608

[15] Hazelton MS , Sheehy PA , Bosward KL , Parker AM , Morton JM , Dwyer CJ , et al. Shedding of *Mycoplasma bovis* and antibody responses in cows recently diagnosed with clinical infection. J Dairy Sci. 2018;101:584‒589. 29055548 10.3168/jds.2017-13512

